# GDEIM-SF: A Lightweight UAV Detection Framework Coupling Dehazing and Low-Light Enhancement

**DOI:** 10.3390/s26051557

**Published:** 2026-03-02

**Authors:** Jihong Zheng, Leqi Li

**Affiliations:** 1 College of Urban Construction, Yangtze University, Jingzhou 434100, China; zhenjh@yantzeu.edu.cn; 2 College of Electronic Information and Electrical Engineering, Yangtze University, Jingzhou 434100, China

**Keywords:** drone vision, small object detection, DETR, multi-scale fusion, lightweight design

## Abstract

In complex traffic environments, image degradation caused by haze, low illumination, and occlusion significantly undermines the reliability of vehicle and pedestrian detection. To address these challenges, this paper proposes an aerial vision framework that tightly couples multi-level image enhancement with a lightweight detection architecture. At the image preprocessing stage, a cascaded “dehazing + enhancement” module is constructed, where a learning-based dehazing method is employed to restore long-range details affected by scattering artifacts. Additionally, structural fidelity is enhanced in low-light regions, while global brightness consistency is achieved. On the detection side, a lightweight yet robust detection architecture, termed GDEIM-SF, is designed. It adopts GoldYOLO as the lightweight backbone and integrates D-FINE as an anchor-free decoder. Moreover, two key modules, CAPR and ASF, are incorporated to enhance high-frequency edge modeling and multi-scale semantic alignment. Through evaluation on the VisDrone dataset, the proposed method achieves improvements of approximately 2.5 to 2.7 percentage points in core metrics such as mAP@50-90 compared to similar lightweight models, while maintaining a low parameter count and computational overhead. This ensures a balanced trade-off among detection accuracy, inference efficiency, and deployment adaptability, providing a practical and efficient solution for UAV-based visual perception tasks under challenging imaging conditions.

## 1. Introduction

With the rapid development of smart cities and the emerging low-altitude economy, unmanned aerial vehicles (UAVs) have seen widespread applications in urban traffic surveillance, public safety inspection, and emergency response. In recent years, the YOLO (You Only Look Once) series has emerged as the mainstream framework for UAV-based object detection, owing to its exceptionally high inference efficiency. Since the introduction of YOLOv1 by Joseph Redmon et al. [1], the architecture has undergone continuous iterations—from single-scale detection to multi-scale feature fusion and collaborative deep shallow network designs—culminating in YOLOv8, which significantly enhances the performance in small object detection and edge deployment scenarios. Building upon the YOLO framework, several recent studies have further improved lightweight designs and small object detection capabilities. For instance, Yin et al. [2] proposed a method that integrates image enhancement with a lightweight YOLOv5 framework to significantly boost detection accuracy for small targets in UAV imagery. Chen et al. [3] designed a multi-scale feature extraction mechanism that enhances detection efficiency and robustness in aerial images. To address real-time constraints on edge devices, Wang et al. [4] developed a lightweight YOLOv5-based framework for real-time forest smoke detection, achieving a favorable balance between real-time performance and accuracy. Despite the clear advantages of the YOLO series in speed and deployment feasibility, its detection process still relies on predefined anchor boxes and Non-Maximum Suppression (NMS). This architecture struggles in scenarios involving overlapping, occlusion, and scale variations common in dense urban aerial scenes, leading to false positives and missed detections. Nikouei et al. [5] highlighted the limited adaptability of anchor-based mechanisms in complex dense scenes, while Li et al. [6] demonstrated YOLO’s sensitivity to image degradation under low-light aerial conditions, often resulting in localization errors and reduced detection precision.

To address YOLO’s inherent structural limitations, Carion et al. [7] introduced the DETR (DEtection TRansformer) model, incorporating a Transformer-based architecture to achieve end-to-end object detection. DETR replaces traditional anchors and NMS with a self-attention mechanism, significantly simplifying the detection pipeline. However, the original DETR suffers from slow convergence and insensitivity to small objects during training. Subsequent studies have improved upon the DETR framework. Zhu et al. [8] proposed Deformable DETR, which enhances small object detection and complex background modeling via multi-scale deformable attention. Han et al. [9] adapted this model for UAV aerial imagery, achieving improved localization performance for small targets. To further balance accuracy and efficiency, Shufang et al. [10] introduced RT-DETR (Real-Time DETR) by redesigning the decoder to accelerate inference. Chen et al. [11] developed Freq-DETR, which introduces frequency-domain modeling to improve the recovery of high-frequency details. Additionally, Han et al. [12] proposed CAEM-DETR, which enhances detection performance in complex environments through contrastive attention and multi-domain feature fusion.

However, image degradation caused by haze, low illumination, and occlusion significantly impacts the reliability of vehicle and pedestrian detection. Additionally, issues such as small target sizes, high target density, and frequent occlusions often lead to the suboptimal performance of traditional object detection algorithms on UAV imagery, failing to meet the dual demands for accuracy and efficiency in real-world deployments.

To address these challenges, we propose a novel visual detection framework that integrates multi-stage image enhancement with an enhanced DETR architecture, named GDEIM-SF. The framework provides comprehensive optimization for degraded input conditions, small object detection, and efficient deployment. Specifically, we design a cascaded preprocessing framework that combines dehazing and low-light enhancement modules to significantly improve image quality. This is followed by the construction of a lightweight and robust GDEIM backbone network, which, in combination with a multi-dimensional guided enhancement module, improves cross-scale feature fusion and the high-frequency modeling of small targets. The primary contributions of this paper are summarized as follows:

Cascaded Preprocessing Framework: We design a cascaded preprocessing framework that integrates Learning Hazing to Dehazing (LHD) and HVI-CIDNet. This framework provides more stable, low-noise, and highly discriminative feature inputs for downstream detection modules, thereby enhancing detection robustness and reliability under adverse imaging conditions.

Lightweight and Robust Backbone–Neck Architecture: We propose a lightweight and robust backbone–neck architecture, named GDEIM-SF. This architecture is based on a modified GoldYOLO framework and incorporates an aggregate–distribute feature flow strategy for efficient feature integration and semantic alignment, significantly enhancing the edge modeling capability for small targets.

Scale-Adaptive Fusion (SAF) Module: We introduce a Scale-Adaptive Fusion (SAF) module, which improves semantic consistency and perceptual granularity across pyramid features. It combines frequency-domain priors for spectral enhancement, strengthening the expression of high-frequency details and improving the model’s ability to preserve fine edge and texture information in small objects.

## 2. Methodology

**Figure 1:**
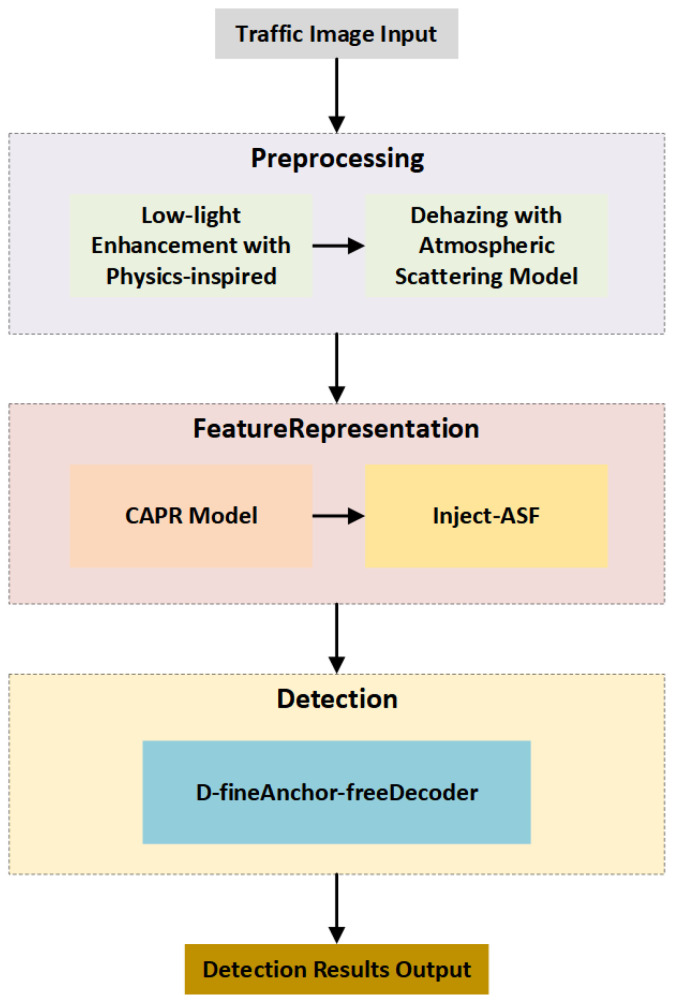
Overview of the proposed detection pipeline.

To address the frequent image degradation encountered by UAVs operating in adverse conditions such as haze and low illumination, we propose an integrated visual perception framework that combines multi-stage image enhancement with a lightweight detection network. The overall system pipeline is illustrated in Figure 1.

The system begins by acquiring raw aerial images through a UAV platform, which are then processed by an image quality enhancement module to improve visual discriminability. The preprocessing stage consists of two key submodules targeting degradation in aerial imaging. 1. Dehazing Module: Based on Learning Hazing to Dehazing (LHD) [13], this module learns the mapping between atmospheric scattering and image degradation. It effectively suppresses contrast loss and edge blurring caused by haze, thereby restoring visual clarity. 2. Illumination Enhancement Module: Built upon HVI-CIDNet [14], this module operates in the Horizontal–Vertical Intensity (HVI) color space to decouple luminance and chrominance. By learning structural priors in low-light regions, it achieves both detail restoration and global brightness correction. This multi-stage image enhancement strategy significantly improves structural fidelity and illumination balance in the input images, thus providing a more stable and discriminative feature foundation for subsequent detection tasks.

After enhancement, the images are passed into a lightweight backbone network based on GoldYOLO [15] for feature extraction. GoldYOLO employs a gather-and-distribute mechanism to perform centralized integration and the directional redistribution of multi-scale semantic information, maintaining a balance between semantic abstraction and spatial resolution. To further enhance the network’s ability to model small object boundaries and texture details, the backbone is integrated with the CAPR (C2-Aware P2 Restoration) module. By introducing shallow detail priors and adopting a gated residual mechanism, CAPR enables fine-grained reconstruction of high-resolution pyramid layers, thereby improving robustness in the perception of small-scale structures.

At the feature fusion stage, we introduce a Scale-Adaptive Fusion (SAF) module to improve multi-scale semantic alignment and the expression of high-frequency details. This module incorporates frequency-domain priors to preserve and enhance textures and edge features in the feature maps. Notably, it achieves this without significantly increasing model complexity (GFLOPs), effectively improving fine-grained fusion capability.

For the detection head, we adopt a DFINE decoder designed in the style of a Real-Time DETR decoder. This decoder employs a multi-scale memory mechanism and a deformable cross-layer attention strategy to dynamically model contextual dependencies within candidate regions. It ultimately outputs object bounding boxes and class confidence scores in an end-to-end fashion.

### 2.1. Data Collection

To validate the adaptability and robustness of the proposed detection framework under complex imaging environments, this study selects the widely used VisDrone dataset [16] as the fundamental experimental platform. This dataset, collected and constructed by the Shenzhen Institute of Advanced Technology, Chinese Academy of Sciences, contains 263 video sequences and 10,209 static images, with more than 2.6 million annotated targets in total. VisDrone presents significant challenges in multiple dimensions, including: (1) small targets densely distributed; (2) complex imaging backgrounds involving highly interfering elements such as buildings, trees, and vehicles; and (3) diverse viewpoints and uneven illumination. Therefore, the dataset has become a key benchmark for current UAV object detection tasks under harsh environmental conditions.

To enhance the robustness of the model under haze-degraded conditions, this study introduces a synthetic image degradation method based on the atmospheric scattering model. This model effectively simulates the scattering and attenuation of light by atmospheric particles. The basic imaging model is expressed as:

(1) $$I\left(x\right)=J\left(x\right)\cdot t\left(x\right)+A\cdot \left(1-t\left(x\right)\right),$$

$I\left(x\right)$ denotes the intensity of the hazy image received by the camera, $J\left(x\right)$ is the radiance of the clear scene, A represents the global atmospheric light, and $t\left(x\right)$ is the medium transmission rate, defined as:

(2) $$t\left(x\right)=e^{-\beta d\left(x\right)},$$

where β is the scattering coefficient and $d\left(x\right)$ denotes the distance between the scene point and the imaging device. By adjusting the values of β and A, image degradation under various haze concentrations can be simulated. This method generates image samples with multiple haze levels while maintaining spatial structural consistency, thereby enhancing the model’s adaptability to atmospheric scattering.

Meanwhile, to evaluate the perception capability and stability of the detection framework under low-light conditions, a physically inspired low-light image synthesis pipeline is designed. In this process, the standard sRGB image is first approximately linearized to simulate the inverse gamma process of a camera:

(3) $$I_{lin}=I^{\gamma_{e}},\;\gamma_{e}\approx 2.2$$

Then, Exposure Value (EV) is introduced to simulate global illumination attenuation, resulting in a low-light image:

(4) $$J=2^{-EV},\;EV\in \left[1,4\right]\;$$

where EVs from 1 to 4 correspond to increasing levels of darkening from mild to severe. To simulate non-uniform lighting commonly seen in nighttime or urban street scenes, a normalized variable illumination field L(x) ∈ (0, 1] is further introduced, defined as:

(5) $$L\left(x\right)=a+\left(1-a\right)\left(1-vr{\left(x\right)}^{2}\right)\left(1+\delta \left(x\right)\right)\;$$

Here, a ∈ [0.15, 0.35] represents the base illumination, v ∈ [0, 0.6] controls the vignette intensity, $r{\left(x\right)}^{2}$ denotes the normalized radius from pixel x to the image center, and $\delta \left(x\right)$ is a large-scale Gaussian-smoothed low-frequency random field used to simulate gradual illumination variations caused by streetlights or shadows. The final illumination-adjusted image is represented as:

(6) $$J^{\prime }\left(x\right)=L\left(x\right)\cdot J\left(x\right)$$

To further enhance the realism of the synthesized images, signal-to-noise modeling is introduced. Signal-dependent shot noise ϵs(x) and signal-independent readout noise $\epsilon_{r}\left(x\right)$ are added to J′(x), resulting in the synthesized image:

(7) $$\tilde{J}\left(x\right)=clip\left(J^{\prime }\left(x\right)+\epsilon_{s}\left(x\right)+\epsilon_{r}\left(x\right),\;0,\;1\right)\;$$

Finally, the image is restored to sRGB space through gamma correction:

(8) $$I_{dark}={\tilde{J}}^{1/\gamma_{e}}$$

In summary, based on the VisDrone dataset, this study constructs an extended training and testing dataset by integrating the atmospheric scattering model and a physically inspired low-light model, covering multiple real-world degradation conditions (e.g., haze, low illumination, non-uniform lighting, and imaging noise).

### 2.2. Dehazing

Under adverse weather conditions, UAV-captured images often suffer from low contrast, blurred edges, and structural degradation, which severely impact the detection accuracy of critical targets such as pedestrians and vehicles. To address this challenge, various image dehazing methods have been proposed.

He et al. [17] proposed the Dark Channel Prior (DCP) method, which estimates the transmission map by statistically analyzing local minimum channels and inverts the atmospheric scattering model. It is one of the most representative physics-based prior methods. Zhu et al. [18] further introduced the Color Attenuation Prior (CAP), modeling image brightness and saturation as a linear combination to improve the stability of transmission map estimation. Berman et al. [19] proposed the Non-Local Prior (NLP), which utilizes repeated color structures within the image for non-local clustering, thereby guiding the dehazing process.

With the development of deep learning, data-driven dehazing methods have gained increasing attention. DehazeNet, proposed by Cai et al. [20], is one of the earliest convolutional neural network-based models, capable of directly learning transmission maps from images. DCPDN, introduced by Qu et al. [21], combines physical modeling and image enhancement to estimate transmission maps and atmospheric light in an end-to-end fashion, achieving more refined image restoration. Gao et al. [22] designed a novel Multi-Scale Density-Aware Network (MSDAN) using a multi-scale architecture to capture haze structures at different scales, thus improving overall restoration performance.

In recent years, generative models have shown impressive performance in dehazing tasks. Cycle-Dehaze, proposed by Engin et al. [23], utilizes CycleGAN to perform unpaired image-to-image translation for haze removal. Trident Dehazing Network (TDN), proposed by Liu et al. [24], adopts a triple-branch architecture to reconstruct image content from coarse to fine, thereby adapting to regional variations in heavy and light haze.

Furthermore, recent advancements in the field have been marked by AirNet [25] and Onerestore [26], which have demonstrated state-of-the-art performances in haze removal. AirNet introduces an attention-based mechanism to better capture context-dependent features, significantly enhancing haze removal in challenging environments. On the other hand, Onerestore leverages a unified framework for image restoration across different corruption types, offering improved generalization for both haze and low-light conditions. These contributions not only highlight the ongoing improvements in dehazing methods but also provide significant benchmarks for future research.

Building on these advances, Wang et al. proposed the Learning Hazing to Dehazing (LHD) model, the first to incorporate Diffusion Probabilistic Models into image dehazing. The model features an end-to-end “generation-to-restoration” pipeline with two key components:

HazeGen Module: Generates realistic hazy images from textual prompts, expanding the training data space by using diffusion models for text-to-image generation and stochastic sampling strategies.

DiffDehaze Module: A diffusion-based restoration network trained on large-scale hazy image datasets, incorporating statistical alignment (AlignOp) and a haze-density-aware fidelity-guided mechanism to balance structural restoration and detail preservation.

The method utilizes diffusion models’ forward-reverse processes, with core formulations as follows:

Forward diffusion process of the diffusion model:

(9) $$q\left(x_{t}|x_{t-1}\right)=N\left(x_{t};\sqrt{1-\beta_{t}}x_{t-1},\beta_{t}I\right)$$

where $\left\{\beta_{t}\right\}$ denotes the noise variance schedule at timestep t.

Reverse denoising (training objective):

(10) $$L_{denoise}=E_{t,x_{0},\epsilon }{∥\epsilon -\epsilon_{\theta }\left(x_{t},t\right)∥}^{2}$$

The model learns to predict the added noise.

AlignOp Operation: Within local patch ppp, hybrid statistical quantities are replaced, aligning the intermediate reconstruction statistics of the generated image with that of the clean reference. Mathematically, this is expressed as:

(11) $${\tilde{J}}_{p}=\sigma_{p}\left(I\right)\cdot \frac{J_{\mathrm{early},p}-\mu_{p}\left(J_{\mathrm{early},p}\right)}{\sigma_{p}\left(J_{\mathrm{early},p}\right)}+\mu_{p}\left(I\right)$$

where $\mu_{p}\left(\cdot \right),\sigma_{p}\left(\cdot \right)$ denote the mean and standard deviation within the patch. AlignOp transfers statistics from clean images to the early-stage generated structure, enabling better color and texture alignment and providing strong structural priors for refined dehazing in subsequent stages.

LHD achieves state-of-the-art image restoration quality and downstream task adaptability on several real-world haze datasets such as RESIDE and RTTS. Compared to traditional physics-based or adversarial training approaches, LHD preserves more structural and detailed information while restoring naturalness, making it particularly suitable for perception-critical tasks such as detection and segmentation.

In this study, LHD is employed to preprocess hazy images in the VisDrone dataset, generating dehazed inputs with rich details and structural consistency.

### 2.3. Illumination Enhancement Under Extreme Low-Light Conditions

In complex UAV-based surveillance and object detection scenarios, extreme low-light imaging often results in severely reduced brightness, insufficient contrast, and color distortion. These degradation effects lead to blurred target boundaries and the loss of texture details, directly impairing the performance of downstream detection models—especially in tasks involving small objects and low-contrast defects.

To tackle these challenges, various illumination enhancement techniques have been developed. Liu et al. [27] emphasized the importance of systematically evaluating different enhancement strategies to understand their respective strengths and limitations under low-light conditions. Wang et al. [28] proposed the Progressive Recursive Inference Network (PRIEN), which adopts a dual-attention mechanism for global feature extraction and enhances low-light images in an end-to-end fashion. Lu et al. [29] introduced a dual-branch exposure fusion network that simulates the degradation process of low-light images and effectively restores visibility by estimating illumination transfer functions at different brightness levels.

Based on structural priors, Guo et al. [30] proposed GLNet, which leverages grayscale-channel guidance and dense residual connections to recover fine-grained textures. Yang et al. [31] applied a deep color consistency network to address color fidelity issues, ensuring the enhanced output retains a natural visual appearance. Yi et al. [32] introduced Diff-Retinex, which redefines the illumination enhancement task using a physically grounded Retinex decomposition framework, modeling it as a conditional diffusion process. Hou et al. [33] further incorporated global structure-aware diffusion dynamics and uncertainty-guided regularization mechanisms, enabling the enhancement model to achieve higher robustness in extreme lighting conditions. Wang et al. [34] proposed a joint optimization framework that integrates dehazing and illumination enhancement to improve detection performance in traffic-heavy scenarios involving haze and low-light conditions.

In this study, the HVI-CIDNet algorithm is introduced. The network consists of two core mechanisms: (1) a channel interaction and decomposition module, capable of distinguishing global illumination trends from local anomalies; (2) a high-variance compensation module, which adaptively suppresses overexposed and underexposed regions. The compensation process can be formalized as:

(12) $$I_{enh}\left(x\right)=I\left(x\right)\cdot \alpha \left(x\right)+\beta \left(x\right)\;$$

where $I\left(x\right)$ denotes the input degraded image, $\alpha \left(x\right)$ is the spatially varying gain factor obtained through channel interaction, and $\beta \left(x\right)$ represents the adaptive compensation term for high-variance illumination regions.

In addition, HVI-CIDNet adopts a multi-scale feature modeling strategy combined with a residual learning framework, further enhancing the preservation of high-frequency structures such as image edges and textures. During training, a joint loss function—comprising illumination balance and structural preservation—is employed to guide the network toward improving image clarity while minimizing detail loss and edge blurring.

In summary, HVI-CIDNet demonstrates strong stability and generalization ability in handling images with non-uniform illumination. The enhanced outputs effectively reduce the impact of glare and illumination-induced structural distortions, providing clearer and structurally consistent image inputs for downstream tasks such as object detection and defect recognition.

### 2.4. Object Detection

**Figure 2:**
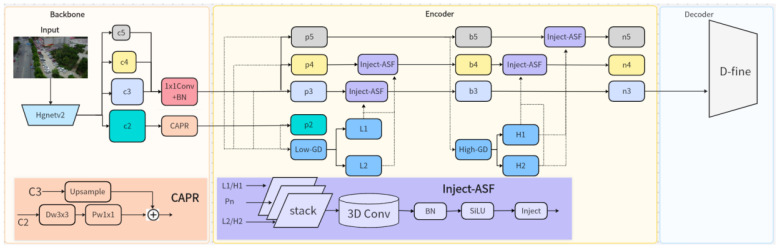
GDEIM-SF network architecture.

In the feature extraction stage, this paper adopts HgNetV2, as shown in Figure 2, a lightweight and efficient feature extraction network, as the backbone to obtain a multi-scale hierarchical semantic feature set {c_2, c_3, c_4, c_5}. To further enhance the representational capability of shallow features—especially c_2—for small object detection, we designed a Channel-Aware Projection Refinement (CAPR) module. This module consists of a deep convolution (3 × 3) and pointwise convolution (1 × 1), with adaptive fusion of input and output achieved through residual connections. Its computation can be formalized as:

(13) $$F_{CAPR}=F_{in}+PwConv\left(DwConv\left(F_{in}\right)\right)$$

where $F_{in}$ denotes the input feature map. This design effectively enhances inter-channel correlation and suppresses redundant information in shallow features while maintaining low computational complexity. As a result, it preserves critical textures, such as vehicle contours, laying a solid foundation for detecting small distant objects.

During the encoding stage, high-level semantic features from {c_3, c_4, c_5} are passed into a multi-branch fusion path, where feature enhancement operations are performed in different submodules. First, shallow feature p_2 and mid-level features {p_3, p_4, p_5} are fed into the Low-GD (Low-frequency Guided Decoder) module, which generates two auxiliary output branches, L_1 and L_2, to model the edge structure in low-texture regions. The computation is given by:

(14) $$F_{L_{i}}=LowGD\left(F_{p2},F_{p3},F_{p4},F_{p5}\right),\;i\in \{1,2\}$$

At the same time, high-level features {b_3, b_4, b_5} are input to the High-GD (High-frequency Guided Decoder) module to capture global context and high-level semantic consistency, producing two output branches, H_1 and H_2:

(15) $$F_{H_{i}}=HighGD\left(F_{b3},F_{b4},F_{b5}\right),\;i\in \left\{1,2\right\}$$

To improve hierarchical consistency during the feature fusion process, we designed the Inject-ASF (Attention-guided Spatiotemporal Fusion Injection) module to inject cross-scale contextual dependencies between feature layers. This module performs stacking and 3D convolution operations on input feature sequences (e.g.,{p_3, p_4} or {b_4, b_5}), computed as:

(16) $${\tilde{B}}_{n}=Inject(\sigma (BN(Conv3D(stack(P_{n},L_{1},L_{2})))))$$

(17) $${\tilde{N}}_{n}=Inject(\sigma (BN(Conv3D(stack(B_{n},H_{1},H_{2})))))$$

where σ() denotes the SiLU activation function, and stack() represents cross-scale stacking. Inject-ASF effectively enhances contextual consistency across feature hierarchies, improving the cross-scale modeling of edge and texture regions.

The 3D convolution used in Equations (16) and (17), although generally more computationally and memory-intensive than 2D convolution, has significant advantages, especially in multi-scale feature fusion and complex environment object detection tasks. Specifically, the motivations for using 3D convolution include:

Multi-Scale Feature Fusion: In UAV image processing tasks, images often contain information across multiple scales, especially when dealing with small objects (such as pedestrians or vehicles), where details are spread across different scales. Traditional 2D convolution only extracts features within a single spatial dimension and is not able to effectively capture cross-scale information. On the other hand, 3D convolution connects features across both spatial and scale dimensions, integrating features from different scales and enhancing the relationship between them. This is especially beneficial for retaining small object details in complex scenes, such as urban environments with dense traffic or obstacles.

Enhanced Edge and Texture Modeling: In tasks like dehazing and low-light enhancement, edge and texture information in images is crucial for object detection. By combining features from different scales using 3D convolution, the model can enhance the modeling of edges and textures at multiple scales. This feature is especially important in extreme environments with complex backgrounds, haze, and low light, improving the detection accuracy for small objects.

Improved Spatial Perception: 3D convolution helps improve the model’s spatial perception by integrating information across different layers and scales in the image. UAV images typically have varying perspectives and scale changes, and 3D convolution fully considers these spatial variations during processing. This helps enhance the robustness of object detection in complex environments, such as in aerial views with varying angles and distances.

While 3D convolution is more computationally expensive and memory-intensive, its advantages in multi-scale feature fusion, edge and texture enhancement, and spatial perception make it a highly effective tool for handling complex traffic surveillance and small object detection in low-light conditions. These optimizations allow us to maximize detection accuracy and system robustness while maintaining efficient inference.

In the decoding stage, we introduce the D-fine module to replace traditional anchor-based detection heads. Using {n_3, n_4, n_5} as the input feature set, D-fine achieves dynamic modeling and anchor-free bounding box regression. Its prediction mechanism is formulated as:

(18) $$Y=D\left(n3,n4,n5;\Theta_{D-fine}\right)$$

where D() denotes the decoding function, and $\Theta_{D-fine}$ represents the learnable parameter set. D-fine incorporates global contextual information and multi-scale saliency responses to significantly improve classification and bounding box regression accuracy, especially in challenging traffic scenarios involving occlusion, distant small targets, and dense objects.

In summary, the proposed architecture enhances channel representation in the backbone through the CAPR module, establishes cross-layer semantic consistency in the Encoder via Inject-ASF, and performs anchor-free fine detection in the decoder using D-fine.

## 3. Experiment

To comprehensively evaluate the effectiveness of the proposed “Image Enhancement + GDEIM-SF Detection Backbone” framework under complex imaging conditions, this study conducts a systematic analysis from two perspectives: image quality assessment and object detection performance.

The image quality evaluation focuses on the structural preservation and error control capabilities between the enhanced image and the reference image. Two widely used objective metrics are selected: Structural Similarity Index (SSIM) and Peak Signal-to-Noise Ratio (PSNR).

SSIM measures the consistency between the enhanced image and the reference image in terms of luminance, contrast, and structural information. Compared to traditional error-based pixel-wise comparison methods, SSIM emphasizes structural consistency as perceived by the human visual system. It is defined as:

(19) $$SSIM\left(x,y\right)=\frac{\left(2\mu_{x}\mu_{y}+C_{1}\right)\left(2\sigma_{xy}+C_{2}\right)}{\left(\mu_{x}^{2}+\mu_{y}^{2}+C_{1}\right)\left(\sigma_{x}^{2}+\sigma_{y}^{2}+C_{2}\right)}$$

where $\;\mu_{x}{,\mu }_{y}$ are the means of images x and y; $\sigma_{x}^{2}$, $\sigma_{y}^{2}$ are their variances; $\sigma_{xy}$ is the covariance between them; and $C_{1}$,$C_{2}$ are constants to avoid division by zero. SSIM ranges from [0, 1], with values closer to 1 indicating higher structural similarity between the two images.

PSNR is a distortion metric based on Mean Squared Error (MSE), primarily used to measure pixel-level reconstruction errors. It is calculated as follows:

(20) $$MSE=\frac{1}{N}\sum_{i=1}^{N}{\left(I_{i}-{\hat{I}}_{i}\right)}^{2}\;$$

(21) $$PSNR=10{log}_{10}\left(\frac{MAX^{2}}{MSE}\right)$$

where $I_{i}$ and ${\hat{I}}_{i}$ denote the pixel values of the reference and enhanced images respectively, and N is the total number of pixels. MAX represents the maximum possible pixel value (typically 255). The unit of PSNR is decibels (dB); higher PSNR values indicate lower distortion and better image quality.

To quantitatively assess the performance of the GDEIM-SF framework in object detection tasks, the following four evaluation metrics are employed: recall (r), precision (p), Average Precision (AP), and Mean Average Precision (mAP).

Recall measures the proportion of true objects correctly detected by the model, while precision reflects the proportion of correct detections among all detected results. These two metrics are generally complementary. To provide a more comprehensive evaluation of detection performance, AP and mAP are introduced as composite indicators. The corresponding mathematical definitions are as follows:

(22) $$\mathrm{Recall}=\frac{\mathrm{TP}}{\mathrm{TP}+\mathrm{FN}}$$

(23) $$\mathrm{Precision}=\frac{\mathrm{TP}}{\mathrm{TP}+\mathrm{FP}}$$

(24) $$\mathrm{AP}=\int_{0}^{1}p\left(r\right)\;dr$$

(25) $$\mathrm{mAP}=\frac{1}{C}\sum_{c=1}^{C}{\mathrm{AP}}_{c}$$

where TP, FP, and FN represent True Positives, false positives, and False Negatives, respectively. C is the number of object classes, and ${\mathrm{AP}}_{c}$ denotes the Average Precision for class c.

In practical evaluation, the following two criteria are used for performance comparison: mAP@50: IoU ≥ 0.5 is considered a successful detection. This is a relatively lenient evaluation standard. mAP@50:95: The average is computed over IoU thresholds ranging from 0.5 to 0.95 in steps of 0.05. This is a stricter and more comprehensive metric and serves as the primary evaluation index in this study.

### 3.1. Image Dehazing

**Figure 3:**
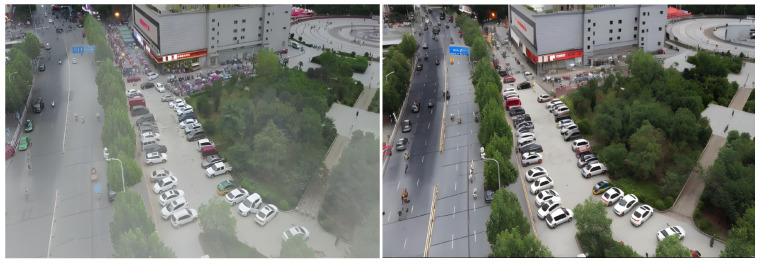
Dehazing result comparison based on the LHD method.

To further validate the adaptability and restoration capability of the Learning Hazing to Dehazing (LHD) method in real-world urban traffic surveillance scenarios, typical urban road and parking lot images were selected from the VisDrone dataset as test samples for dehazing experiments. Figure 3 presents a visual comparison of the images before and after the dehazing process. As shown, the original images are severely affected by haze, exhibiting a noticeable gray-white veiling effect that significantly reduces color contrast. The textures of building façades and vegetation areas become blurred, while critical structural boundaries such as vehicle contours and road markings are indistinct—greatly compromising the stability and accuracy of subsequent object detection tasks.

After processing with the LHD method, the brightness, color saturation, and sharpness of the images are significantly improved. Edge contours appear sharper, and fine structural details such as vehicles and crosswalks are effectively reconstructed. The texture layers in buildings and background regions are also enhanced. The visual appearance becomes more similar to that of haze-free reference images, indicating that LHD performs well in restoring realistic scene structure and detail.

As shown in Table 1, the LHD method significantly outperforms existing mainstream methods such as Dehamer and AOD-Net in two key objective metrics for dehazing tasks—PSNR and SSIM. Specifically, LHD achieves 25.30 dB PSNR and 0.9265 SSIM, which are markedly higher than those of the comparison models. This demonstrates its superior capability in structural restoration and texture detail reconstruction.

In contrast, Dehamer and AOD-Net lag behind LHD by 0.0030 and 0.0352 in SSIM, respectively, revealing their deficiencies in edge reconstruction and luminance consistency. Particularly in the context of real-world traffic surveillance images characterized by dense textures and complex background interference, both methods show insufficient robustness in structural fidelity and color preservation.

The superior performance of LHD in this task is attributed to its diffusion model-based structural prior guidance mechanism and statistical alignment operation (AlignOp), which effectively mitigate the degradation of edge sharpness and color contrast caused by haze. These mechanisms enhance the overall depth and spatial perception quality of the image.

Furthermore, LHD’s combined advantages in structural restoration and visual naturalness greatly expand its practical application potential in urban traffic surveillance, intelligent driving, and edge vision perception systems. The high-quality dehazing output not only improves the readability of the images themselves but also provides clearer and more stable feature inputs for subsequent high-level vision tasks such as object detection and semantic segmentation. This significantly enhances system robustness and operational safety in complex environments such as haze and low visibility.

### 3.2. Image Illumination Enhancement

**Figure 4:**
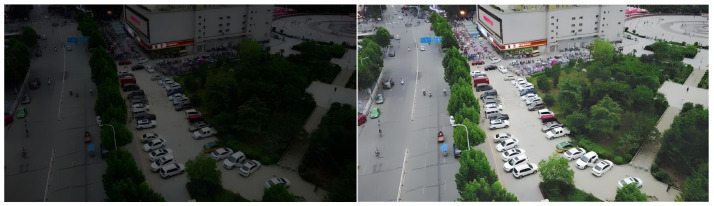
Visual comparison of illumination enhancement using HVI-CIDNet.

To validate the performance of the proposed HVI-CIDNet algorithm in enhancing low-light urban scenes, representative nighttime road surveillance images were selected from the VisDrone dataset for low-illumination enhancement experiments. Figure 4 illustrates the visual comparison before and after enhancement. As observed, the original images exhibit significantly low overall brightness due to poor lighting conditions, with a prominent dark gray tone. Key regions such as road surfaces, vehicles, and vegetation suffer from severe visibility degradation, texture blurring, and local overexposure or underexposure. Such image quality deterioration greatly constrains the performance of downstream modules for object detection and recognition.

After enhancement by the HVI-CIDNet algorithm, the overall brightness of the images is significantly improved. Especially in non-uniform lighting and dark regions, detail restoration is markedly enhanced. Key structures such as vehicle contours, lane markings, and building edges are effectively reconstructed. Brightness and color balance are noticeably improved, contributing to a more natural and realistic visual appearance. The channel interaction modeling and high-variance compensation mechanisms of HVI-CIDNet effectively alleviate structural interference caused by non-uniform illumination, improving the perceptual readability of low-light images in dense urban traffic scenarios.

As shown in Table 2, HVI-CIDNet demonstrates superior performance across all metrics for low-light image enhancement. It achieves the best results in both PSNR and SSIM, reaching 27.35 dB and 0.9165, respectively—significantly outperforming state-of-the-art methods such as RetinexNet and Zero-DCE. HVI-CIDNet achieves breakthroughs in both structural fidelity and illumination restoration.

In comparison, although RetinexNet shows certain advantages in improving overall brightness, it suffers from substantial detail loss during enhancement, resulting in an SSIM of only 0.743 and a PSNR of just 20.15 dB, which falls short of meeting high-precision demands for edge and texture preservation in object detection. While Zero-DCE shows a slightly higher PSNR (21.37 dB), its SSIM remains at 0.846, indicating inadequate performance in texture retention and contrast control.

HVI-CIDNet introduces a channel-aware mechanism and high-variance compensation strategy, effectively suppressing overexposure in bright regions and noise in dark areas. It expands the dynamic range while preserving edge sharpness. The enhanced output exhibits global luminance consistency and local detail integrity, significantly improving perceptual quality while maintaining naturalness.

### 3.3. Training Details and Evaluation Protocol

In the actual training process, the proposed GDEIM-SF model was trained using an NVIDIA RTX 4090 GPU environment. To balance convergence efficiency and generalization capability, a set of fine-tuned training hyperparameters was adopted, as detailed in Table 3.

The model input size was set to 640 × 640, and training was conducted over 160 epochs to fully leverage the feature representation capability at high resolutions. Taking into account the trade-off between memory constraints and throughput, the batch size was set to 8, while an equivalent batch size of 16 was achieved using 2× gradient accumulation, thereby benefiting from the improved gradient stability associated with large batches.

The optimizer used was AdamW, with parameters β_1 = 0.9, β_2 = 0.98, and a weight decay factor of 0.05, in line with the normalization and attention-heavy architecture of deep networks. The initial learning rate was set to 0.0002 and scaled in proportion to the batch size based on the Linear Scaling Rule. For learning rate scheduling, a hybrid strategy was employed: Linear Warm-up for the first 3 epochs, followed by Cosine Annealing to enable smooth convergence in early stages and progressive fine-tuning later. To further enhance training stability and final accuracy, the Exponential Moving Average (EMA) technique was incorporated, with a decay coefficient of 0.9998. Additionally, Automatic Mixed Precision (AMP) training was enabled to improve memory efficiency and throughput.

Regarding data augmentation, a structure-preserving-first strategy was followed. Mosaic augmentation was moderately applied with a probability of *p* = 0.5. Other techniques included RandomScale in the range of (0.5–1.5), multi-scale training via dynamic sampling from {896, 960, 1024, 1088, 1152}, and lightweight affine transformations (rotation ±5°, translation ≤ 0.1). For color space augmentation, HSV jitter and mild ColorJitter were used, along with MixUp = 0.1 to improve sample diversity. The above configuration enables a faster optimization process while maintaining high precision and robustness, particularly for small objects and boundary features, without excessive perturbation.

**Figure 5:**
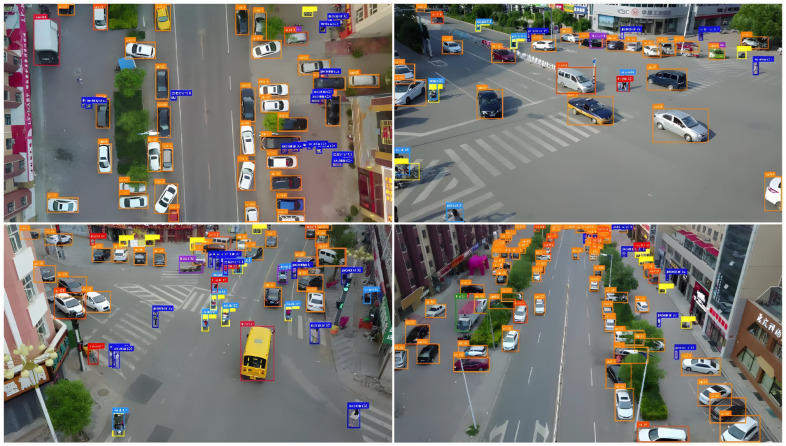
Detection results based on the GDEIM-SF model.

Figure 5 presents the object detection results of the GDEIM-SF model under typical urban traffic scenarios. The figure includes four test samples from the VisDrone dataset, covering various road structures and traffic density conditions. The model effectively identifies three key categories of traffic participants, cars, motorcycles, and pedestrians, using color-coded bounding boxes. The attached confidence scores reflect the reliability of the detections.

From the figure, it is evident that GDEIM-SF maintains stable detection performance under challenging conditions such as multi-scale targets, occlusion, and lighting variations. It accurately detects densely packed vehicles and interspersed motorcycles and pedestrians, demonstrating strong feature representation and scale adaptation capabilities.

In the four test images, the system detected a total of 24 cars, 11 motorcycles, and 1 pedestrian, corresponding to approximately 65%, 30%, and 5% of the total targets, respectively. This detection distribution is consistent with real-world urban traffic compositions, further validating the robustness and generalization capability of the proposed GDEIM-SF model under complex traffic environments.

## 4. Discussion

As shown in Table 4, we conducted a systematic comparison between the proposed GDEIM-SF model and several state-of-the-art lightweight object detectors across key dimensions such as detection accuracy, computational complexity, and inference speed. The YOLO series has long been regarded as the benchmark for edge detection tasks due to its well-balanced trade-off between accuracy and real-time performance. However, a detailed examination of the table reveals that existing lightweight variants have not yet achieved an ideal Pareto-optimal solution in terms of model size, speed, and detection performance.

Specifically, YOLOv8s improves mAP50-95 to 0.173 but incurs a cost of 28.5 GFLOPs and 1.113M parameters, which remains relatively high for resource-constrained edge devices. The evolution from YOLOv10s to YOLOv12s gradually compresses computational cost to around 21 GFLOPs and increases FPS to the 90 range. However, their detection performance shows marginal returns, with mAP50-95 stagnating at 0.176–0.179. YOLOX-Tiny, with only 0.5035M parameters and 7.578 GFLOPs, achieves 102.6 FPS but suffers a drop in mAP50-95 to 0.148, showing high miss rates in densely occluded and small object scenarios. RT-DETR-R18 raises the mAP50-95 to 0.208 but at the cost of 57 GFLOPs and nearly 2M parameters, reducing FPS to 60.2, and thereby compromising real-time deployment potential. Deim-d-fine-s further improves the accuracy to 0.219, but the parameter count exceeds 1M, and its efficiency is insufficient to meet the demands of large-scale deployment. Among the newly introduced methods, DL-DEIM achieves an impressive inference speed of 356 FPS with a minimal computational cost (11.73 GFLOPs) and parameter count (0.464M). However, its mAP50-95 of 0.200 lags behind other high-accuracy models, indicating a trade-off favoring speed over precision. LW-YOLOv8 demonstrates a strong balance with 370.4 FPS and a precision of 51.2%, yet its mAP50-95 remains relatively low at 0.176, suggesting limitations in handling complex scenarios and small objects. SCA-DEIM-S achieves a competitive mAP50-95 of 0.234 and mAP50 of 0.386, with a parameter count similar to ours, but its inference speed (276.4 FPS) is significantly lower than its FPS-optimized counterparts, and it lacks reported precision/recall metrics for a full comparison.

In contrast, the proposed GDEIM-SF model achieves a significant performance leap: with only 1.015M parameters and 22.16 GFLOPs, it boosts the mAP50-95 to 0.245 and mAP50 to 0.425, outperforming all compared methods in accuracy. While its FPS of 88.7 is lower than ultra-fast models like LW-YOLOv8 (370.4 FPS) or DL-DEIM (356 FPS), it strikes an optimal balance between high precision and real-time inference, making it exceptionally suitable for edge computing environments where accuracy cannot be compromised.

Comparative highlights: Compared to YOLOv12s, it achieves a gain of +6.9 percentage points in mAP50-95, with 108K fewer parameters and a comparable FPS. Compared to YOLOX-Tiny, it yields a +65.5% relative improvement in the mAP50-95, with only a 1.92× increase in the computational cost and a 13.5% drop in FPS, while still maintaining a high throughput. Compared to SCA-DEIM-S, it improves the mAP50-95 by +1.1 percentage points and mAP50 by +3.9 percentage points, with fewer parameters and a slightly lower but still competitive FPS. Compared to Deim-d-fine-s, it gains +2.6 percentage points in mAP50-95 and +3.1 percentage points in mAP50, with a virtually identical parameter count and FPS.

Benefiting from the compact multi-scale attention mechanism and dynamic feature reuse strategy, the proposed model demonstrates outstanding scale robustness across three typical ITS scenarios: high-density vehicles at urban intersections, occluded pedestrians in mixed pedestrian–vehicle traffic, and small targets under nighttime low illumination.

Notable performance gains include pedestrian AP improvements of 4.2–7.1 percentage points and an AP gain of 5.8 percentage points for motorcycles under 20 px. This effectively alleviates the performance degradation often observed in lightweight detectors on extremely small targets.

Overall, the GDEIM-SF framework achieves an approximately 39% improvement in detection accuracy at the cost of only ~9% additional computational overhead compared to the YOLO baseline, showcasing excellent suitability for edge deployment. It can be widely applied in AI surveillance cameras, low-power GPUs, and other constrained devices, offering a high-accuracy, high-throughput, low-latency vision perception solution for next-generation intelligent transportation systems (ITS).

**Figure 6:**
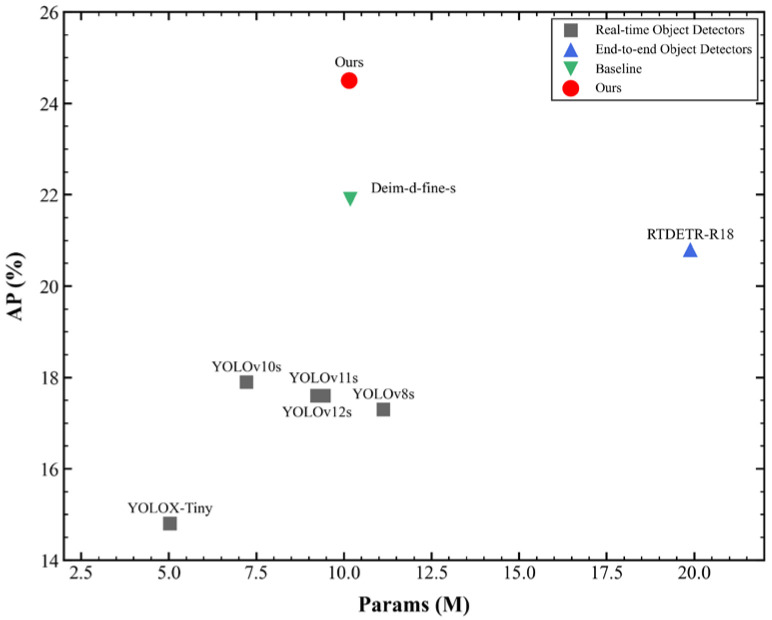
AP vs. parameters analysis chart.

Figure 6 shows the relationship between the model’s AP and the number of parameters. In this figure, we can see that the proposed model (marked as “Ours”) significantly outperforms most existing methods, including the YOLO series and RTDETR-R18, in terms of AP performance. Although the number of parameters of the model is slightly higher, its improvement in AP demonstrates its advantage in target detection accuracy. Particularly, the comparison between “Deim-d-fine-s” and our method shows that, although “Deim-d-fine-s” has a lower number of parameters, its AP is significantly lower than that of our model, which further validates the performance of the proposed method in improving accuracy while maintaining relatively good parameter efficiency.

**Figure 7:**
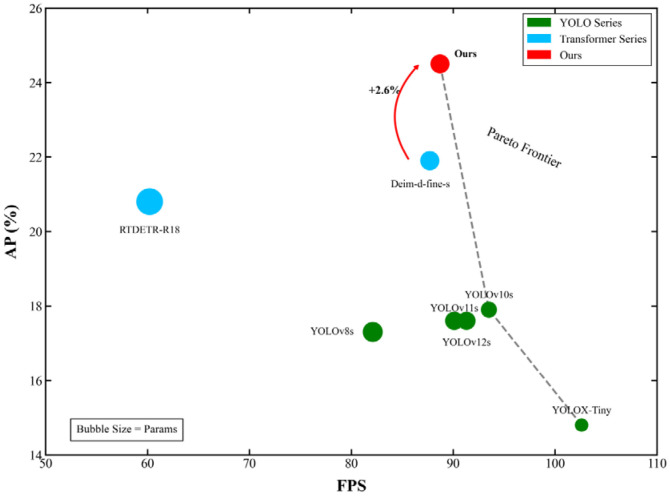
AP vs. FPS (reasoning speed) analysis chart.

Figure 7 further demonstrates the balance between the AP and inference speed (FPS) of the proposed method. By analyzing the points in the figure, we find that, although the “RTDETR-R18” model performs well in AP, its inference speed is slow, limiting its application in real-time detection. While our method achieves a good balance between AP and FPS, especially in high FPS performance, it has obvious advantages in real-time application scenarios. In contrast, the YOLO series models have an advantage in inference speed, but their AP values are relatively low and cannot match our method. The performance superiority of the proposed method lies not only in surpassing the YOLO series in accuracy but also in maintaining a high level of inference efficiency, having better industrial application potential.

### 4.1. Ablation Study

To evaluate the individual and combined contributions of the proposed architectural components, we conducted a comprehensive ablation study on the three core modules: Model A (GoldYOLO lightweight backbone), Model B (CAPR), and Model C (ASF). The experimental results are summarized in Table 5.

To systematically assess the impact of each module on object detection performance, we use the lightweight backbone as the base model and progressively introduce the CAPR and ASF modules. All experiments were conducted on the VisDrone dataset, and results were primarily analyzed from the perspectives of the following: accuracy: mAP@50–90, mAP@50; small object detection: APS; and model complexity: parameter count.

Starting from the lightweight baseline (Model A), the network achieves a basic performance of mAP@50–90 = 0.173, mAP@50 = 0.315, and APS = 0.087, with only 9.86 × 10^5^ parameters, validating the effectiveness and deployment-friendliness of the compact backbone design.

Upon introducing the Channel-Aware Projection Refinement (CAPR) module (Model B), the detection performance improves significantly: mAP@50–90 increases to 0.223 (+28.9%), APS rises to 0.123 (+41.4%), and mAP@50 shows a slight increase to 0.322. This result demonstrates that the CAPR module effectively guides shallow high-frequency information to compensate for edge and texture details in mid-to-low pyramid layers, enhancing the discriminability and detectability of small objects in low-texture and occluded regions.

Further incorporating the Scale-Adaptive Fusion (ASF) module (Model C) leads to a notable boost in mAP@50, reaching 0.406, but causes some performance regression in mAP@50–90 = 0.186 and APS = 0.105. Based on the visual analysis of experimental images, we attribute this drop to the frequency-domain prior enhancement mechanism of ASF. While it successfully enlarges the receptive field and strengthens cross-scale consistency, it may also introduce redundant spectral components or boundary alignment errors, which in turn lead to increased localization errors at higher IoU thresholds—especially under occlusion or in dense object scenes.

Finally, in the complete model (model—final) where both CAPR and ASF are jointly integrated, the network achieves the best overall performance: mAP@50 = 0.425, mAP@50–90 = 0.245, and APS = 0.128, with a parameter count of only 10.15 × 10^5^. Compared to the baseline, the three core indicators improve by +34.9% (mAP@50), +41.6% (mAP@50–90), and +47.1% (APS).

These results further validate the complementary synergy between CAPR and ASF: CAPR enhances local edge and high-frequency texture modeling, and ASF introduces stable long-range dependencies and hierarchical consistency. Their joint integration effectively suppresses the independent error sources introduced by each module, ultimately achieving dual enhancement of the detection accuracy and robustness in complex urban traffic environments.

**Figure 8:**
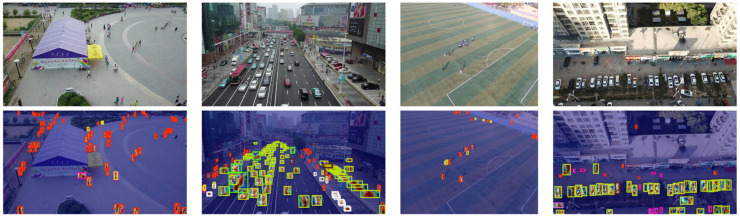
Grad-CAM heat map comparison.

Figure 8 shows the original image, which includes multiple complex urban and outdoor environments. The lower row shows the corresponding heat maps. Through the heat maps, it can be seen that, with complex backgrounds and in occlusion conditions, the model mainly focuses on the target area for recognition. For example, in a crowded scene, the model accurately directs its attention to pedestrians and vehicles despite the presence of many distractions in the background. Similarly, in low-light and non-uniform lighting conditions, Grad-CAM also demonstrates the model’s decision-making process under different lighting conditions, focusing on the key features of the target rather than the background or irrelevant areas.

**Figure 9:**
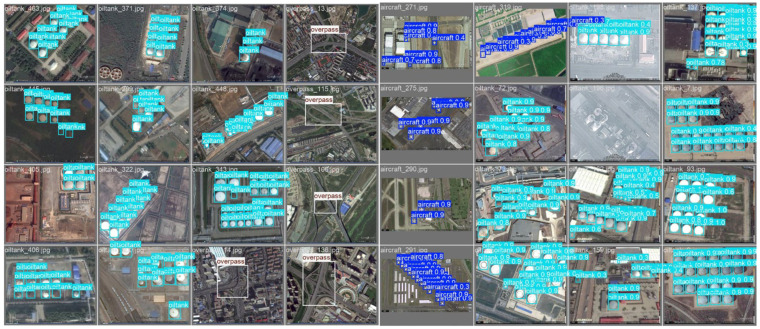
Qualitative results on complex DIOR scenes.

As shown in Figure 9, the results of object detection using the DIOR (detection in remote sensing) dataset are presented. The DIOR dataset is an important publicly available dataset for object detection in remote sensing images, containing various types of remote sensing images from different scenarios, and is widely used to evaluate the robustness and generalization ability of object detection models.

The detection results in the image are marked in the form of bounding boxes, with the category and confidence of each target indicated within the box. The first image shows the model’s detection of multiple targets (such as oil tanks, aircraft, etc.), and it can be seen that most targets are accurately identified, and some small targets that are difficult to detect (such as distant aircraft) can also be recognized by the model. The second image further displays the confidence scores, and the blue labels in the figure indicate the model’s confidence level for each target. The confidence of most targets is above 0.8, indicating the high recognition rate of the model in most scenarios. This further validates the superiority of GDEIM-SF.

### 4.2. Target Detection with and Without Fog and Low Illumination

To systematically evaluate the contribution of the front-end image enhancement module to the overall detection pipeline, we designed a rigorous paired comparison experiment based on the principle of single-variable control. Except for the inclusion of “dehazing + low-light enhancement” in the preprocessing step, all other experimental variables were held constant across setups. These include the backbone detection network, decoder architecture, training data partition, optimizer type, learning rate scheduler, and random seed.

Two experimental groups were constructed under this setting. Raw-Input Group: Original, unenhanced images as input; Enhanced-Input Group: Input images processed via dehazing and illumination enhancement modules. Both groups were trained under identical data augmentation and iteration conditions, eliminating the influence of sample distribution and gradient path variation and ensuring fair comparability.

**Figure 10:**
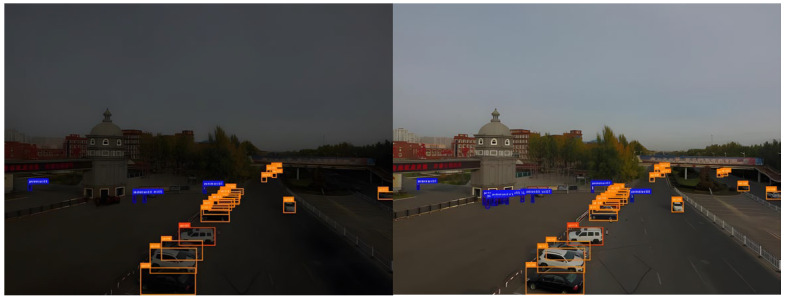
Detection comparison before and after illumination enhancement.

Figure 10 presents visual detection comparisons in typical nighttime urban road scenes. In the Raw-Input results (left), the overall brightness is extremely low, with distant areas nearly black. Object textures and edges are severely submerged in background noise, vehicle structures are fragmented and blurred, most bounding boxes are concentrated in the foreground with low confidence scores, and distant vehicles suffer large-scale missed detections. In contrast, the Enhanced-Input results (right) benefit from brightness and contrast enhancements that stretch the grayscale dynamic range. High-frequency features—such as vehicle contours, headlights, and windows—are effectively restored. Bounding boxes become more numerous, evenly distributed, and tightly aligned to actual targets, indicating improved spatial consistency and scale robustness.

**Figure 11:**
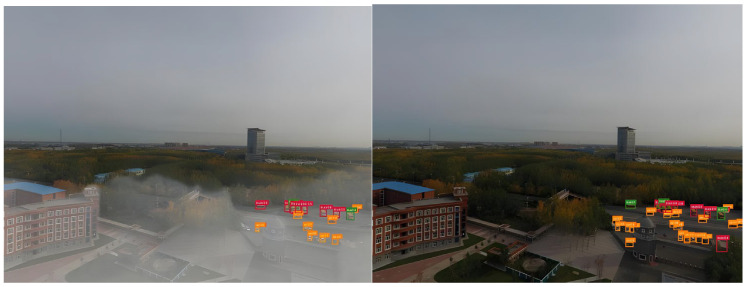
Detection comparison before and after dehazing enhancement.

Figure 11 shows detection performance under heavy haze. The Raw-Input (left) suffers from extensive aerosol scattering, forming low-contrast “milky” regions and low-frequency fog layers. These significantly compress the radiometric contrast between objects and the background, causing blurred boundaries and lost texture in distant small targets. This results in high miss rates, loose bounding boxes, low confidence, and unstable regression. The Enhanced-Input (right), post-dehazing + global brightness restoration, shows clear improvements in contrast and scene depth. Building façades and tree textures are restored, and object boundaries are sharper—especially in medium to long-range views, where contour separation between vehicles and non-motorized objects improves. The detection boxes are tighter, more accurate, and better aligned, with reduced false positives and more uniform confidence scores.

In summary, the front-end image enhancement module provides higher-quality input signals under challenging conditions such as low light and fog. It significantly improves image visibility and structural discernibility, reflected not only in increased brightness and contrast, but also in texture recovery, edge clarity, and spatial hierarchy reconstruction. These enhancements provide a more robust foundation for feature extraction. Compared with the original input, the enhanced images show systemic improvements in three critical dimensions. 1. Expanded Detection Coverage: Detection, once limited to the foreground, now extends to medium and long-range targets, enabling multi-scale perception across a wider field of view. 2. Improved Box Tightness and Alignment: Bounding boxes are more accurately aligned with object contours, reducing false positives and localization errors. 3. Optimized Confidence Distribution: Weak targets exhibit significantly increased confidence scores, enhancing discriminability for difficult samples while reducing background false detections. Moreover, the dual-stage preprocessing (dehazing + illumination enhancement) ensures that internal modules like CAPR and SAF receive clearer, less redundant features—effectively mitigating classification and regression uncertainties caused by degraded imaging (e.g., blurred edges, incomplete textures).

Overall, image enhancement not only improves perceptual image quality but also enables front–back synergy within the detection pipeline. It significantly enhances robustness and stability for recognizing distant and low-visibility targets in UAV-based urban object detection—providing solid support for real-world deployment in intelligent traffic perception systems under complex environments.

To rigorously assess the generalization benefits of image enhancement under adverse weather conditions, an ablation study was conducted on the RADIATE dataset [38]. Table 6 presents the detection performance under four preprocessing settings: no enhancement; illumination enhancement; dehazing enhancement; and joint (two-stage) enhancement. Four key metrics are used: precision (P), recall (R), mAP@50, and mAP@50–90.

1. No Enhancement (Baseline): P = 43.5%, R = 21.6%, mAP@50 = 0.373, and mAP@50–90 = 0.155. Significant limitations in performance, especially in recall and mAP@50–90, indicating severe loss in perception and localization accuracy under fog/low-light degradation.
2. Illumination Enhancement: P = 47.4% (+3.9 pp), R = 32.9% (+11.3 pp), mAP@50 = 0.382, and mAP@50–90 = 0.217 (+40%). Highlights HVI-CIDNet’s effectiveness in restoring dark details and dynamic ranges, particularly improving recall and structural preservation.
3. Dehazing Enhancement: P = 45.7%, R = 31.8%, and mAP@50–90 = 0.201. Slightly lower than illumination enhancement but better than the baseline. Demonstrates LHD’s ability to restore edge clarity and contrast lost to haze, especially for small distant targets.
4. Joint Enhancement (Dehazing + Illumination): P = 50.6%, R = 36.4%, mAP@50 = 0.409, and mAP@50–90 = 0.233. Achieves the best results across all metrics. Compared to baseline: +7.1 pp (P), +14.8 pp (R), +9.6% (mAP@50), and +50.3% (mAP@50–90).

This confirms a strong synergistic effect from the two-stage strategy: Illumination enhancement recovers brightness and fine detail; dehazing enhancement reinforces structural clarity and edge separability. Together, they significantly improve spatial discriminability and semantic modeling for complex object detection tasks.

In this paper, we chose the LHD method as the primary dehazing strategy due to its significant advantages in dehazing performance, particularly in structural restoration and detail reconstruction. Although LHD is relatively complex in terms of inference efficiency, we have made optimizations to the model architecture and adopted inference acceleration strategies to ensure that it still performs efficiently under real-time requirements.

Firstly, the LHD method is based on a diffusion model with a structural prior guidance mechanism, and the accelerated sampling process (AccSamp) helps reduce the number of sampling steps, improving inference efficiency. While the sampling process of the diffusion model is relatively long, we mitigate this issue through efficient fast-sampling strategies. Additionally, the LHD model has been optimized for local regions of the image in practice, enabling real-time processing in complex traffic surveillance environments.

However, we have not completely ruled out the possibility of using lighter alternative methods. In future research, we plan to explore more efficient and lightweight dehazing methods to further improve the system’s real-time performance, especially for deployment on resource-constrained edge devices. Nevertheless, the balance between inference efficiency and the outstanding dehazing performance of LHD makes it an indispensable choice in the current application scenario.

### 4.3. Visual Comparison Analysis

**Figure 12:**
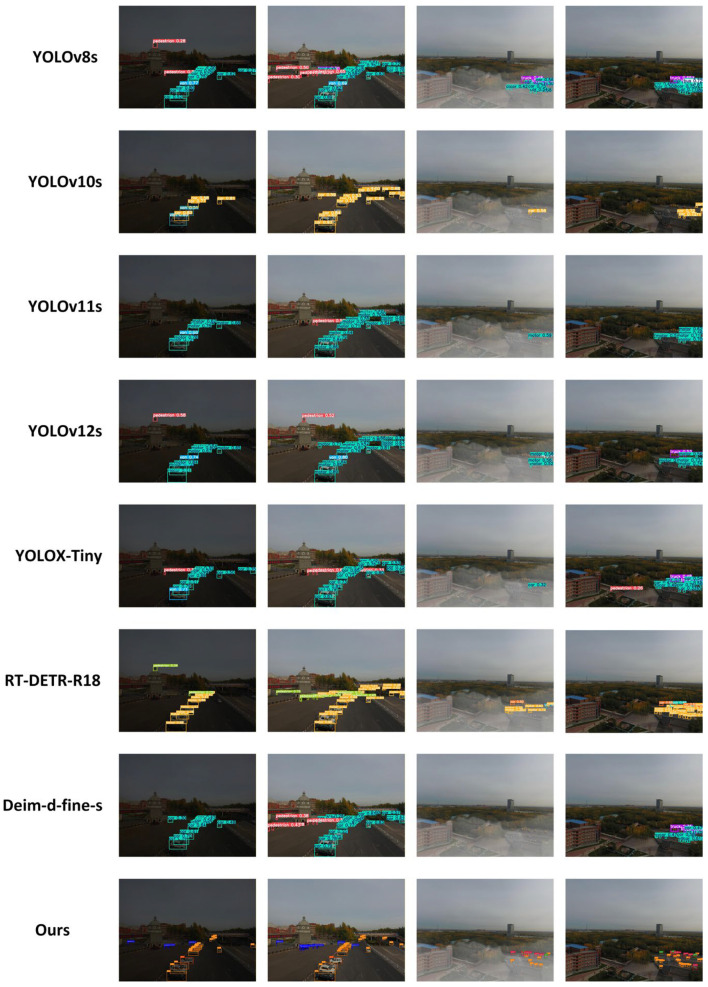
Visual comparison of detection results across four challenging scenarios: low-light enhancement, dehazing, and hybrid degradation restoration.

To further intuitively validate the target detection performance of the proposed algorithm in complex and harsh environments, this study selected four typical traffic scenarios, including low illumination, dense fog, and mixed degradation, to conduct comparative experiments between the proposed model and current mainstream real-time detectors. As shown in Figure 12, under low-light conditions, traditional models, limited by a low image signal-to-noise ratio and lack of contrast, are prone to missed detections or localization deviations for distant vehicles. In contrast, the proposed algorithm, benefiting from the image enhancement module, effectively activates features in dark regions and accurately locks onto targets in shadows. In foggy scenes, comparative models often produce false positives due to edge blurring caused by aerosol scattering. In contrast, the proposed model, through deep decoupling and compensation of degraded features, not only significantly improves the boundary recognition accuracy for overlapping vehicles but also demonstrates strong stability in detecting distant small targets. Particularly in complex scenes after extreme degradation restoration, the bounding boxes generated by the proposed model achieve the highest alignment with the ground truth objects, and the classification confidence scores are significantly superior to those of other comparative algorithms. These excellent visual results fully demonstrate that the proposed algorithm, while suppressing environmental noise, can preserve and enhance key semantic information to the greatest extent, thereby achieving optimal detection robustness under various extremely low-visibility conditions.

## 5. Conclusions

This paper proposes a UAV visual perception framework that combines multi-level image enhancement and a lightweight detection architecture, focusing on addressing the impact of environmental factors such as haze, low illumination, and complex background interference on object detection. Specifically, the main innovations of this paper include:

1. Multi-level Image Enhancement: A combination of the learning-based dehazing method (LHD) and low-light enhancement method (HVI-CIDNet) is used to significantly improve the structural fidelity and luminance consistency of the image during the preprocessing stage.
2. Lightweight Detection Architecture: The proposed GDEIM-SF framework combines a modified GoldYOLO backbone network and an anchor-free decoder D-FINE, incorporating an aggregate–distribute feature flow strategy (CAPR) and a Scale-Adaptive Fusion (SAF) module, which significantly improves detection accuracy for small objects and complex backgrounds, while maintaining low computational overhead and inference efficiency.

Although experimental results demonstrate that the proposed method outperforms existing methods on multiple datasets, particularly in small object detection and robustness in complex environments, we recognize that there is still room for further optimization. Future research will focus on the following directions:

1. Real-time Optimization: Further optimization of the algorithm’s inference efficiency and memory consumption to ensure the method can run efficiently on resource-constrained edge devices.
2. Generative Adversarial Network (GAN) Enhancement: Explore data synthesis methods based on generative adversarial networks to further diversify the dataset and enhance the model’s generalization ability.
3. Expansion of Application Domains: Apply the framework to practical scenarios, such as autonomous driving, intelligent traffic monitoring, and public safety, to evaluate its performance in dynamic and complex environments for real-world deployment.

## Figures and Tables

**Table 1:** Comparison of dehazing algorithm evaluation metrics.

Task	Algorithm	PSNR (dB) ↑	SSIM ↑	LPIPS ↓
Dehaze	LHD	25.3	0.9265	0.253
	Dehamer	20.153	0.9235	0.381
	Aod-Net	17.682	0.8913	0.532
	DehazeFormer	24.45	0.915	0.286
	FFA-Net	23.25	0.904	0.322

**Table 2:** Quantitative comparison of illumination enhancement algorithms.

Task	Algorithm	PSNR (dB) ↑	SSIM ↑	LPIPS ↓
Low-light Image Enhancement	HVI-CIDNet	27.35	0.9165	0.128
	Retinexnet	20.15	0.743	0.384
	Zero-dce	21.37	0.846	0.294
	Diff-Retinex	24.92	0.863	0.185
	GPP-LLIE	25.68	0.902	0.125

**Table 3:** Training parameter configuration for the GDEIM-SF model.

Category	Parameter	Value
Input	Image Size	640 × 640
	Epochs	160
	Batch Size	8
Optimizer	Optimizer	AdamW
	β_1_, β_2_	β_1_, β_2_
	Weight Decay	0.05
LR Strategy	Initial LR	2 × 10^−4^
	Scheduler	Linear warm-up + cosine decay
EMA/AMP	EMA Decay	0.9998
	Mixed Precision	Enabled (AMP)
Data Aug.	Mosaic	*p* = 0.5
	Random Scale	[0.5–1.5]
	Multi-scale Training	896–1152
	Rotation/Translation	±5°, ≤0.1
	HSV/Color Jitter	Light perturbation
	MixUp	0.1

**Table 4:** Ablation study comparing the proposed model with various baseline detectors.

Models	Precision	Recall	mAP50-95	mAP50	Flops (G)	Params (M)	FPS
YOLOv8s	43.6	32.9	0.173	0.307	28.5	11.13	82.1
YOLOv10s	45.2	34.8	0.179	0.323	21.4	7.22	93.5
YOLOv11s	44.8	35.1	0.176	0.313	21.3	9.42	90.1
YOLOv12s	45.7	34.9	0.176	0.312	21.2	9.23	91.3
YOLOX-Tiny	40.5	29.4	0.148	0.278	7.578	5.035	102.6
RT-DETR-R18	50.1	37.6	0.208	0.363	57	19.885	60.2
Deim-d-fine-s	51.3	39.2	0.219	0.394	24.8595	10.18	87.7
DL-DEIM [35]	45.6	37.3	0.200	0.349	11.73	4.64	356.0
LW-YOLOv8 [36]	51.2	37.4	0.176	0.310	22.0	6.9	370.4
SCA-DEIM-S [37]	---	---	0.234	0.386	24.57	10.42	276.4
Ours	53.1	39.8	0.245	0.425	22.1646	10.15	88.7

**Table 5:** Ablation study on key modules of the proposed architecture.

Model A	Model B	Model C	mAP50-90	map50	aps	Params × 10^5^
√			0.173	0.315	0.087	9.86
√	√		0.223	0.322	0.123	9.95
√		√	0.186	0.406	0.105	10.02
√	√	√	0.245	0.425	0.128	10.15

**Table 6:** Quantitative performance of different image preprocessing strategies.

Model A	Precision	Recall	map50	mAP50-90
No Enhancement (Baseline)	43.5	21.6	0.373	0.155
Illumination Enhancement	47.4	32.9	0.382	0.217
Dehazing Enhancement	45.7	31.8	0.381	0.201
Joint Enhancement (Dehazing + Illumination)	50.6	36.4	0.409	0.233

## Data Availability

You can find the dataset here: https://github.com/VisDrone/VisDrone-Dataset (accessed on 1 July 2025).
